# Relationship between retinal fluid and visual acuity in patients with exudative age-related macular degeneration treated with intravitreal aflibercept using a treat-and-extend regimen: subgroup and post-hoc analyses from the ALTAIR study

**DOI:** 10.1007/s00417-021-05293-y

**Published:** 2021-07-20

**Authors:** Masahito Ohji, Annabelle A. Okada, Koji Sasaki, SungChul Charles Moon, Tobias Machewitz, Kanji Takahashi, the ALTAIR Investigators

**Affiliations:** 1 Department of Ophthalmology, Shiga University of Medical Science, Shiga, Japan; 2 Department of Ophthalmology, Kyorin University School of Medicine, Tokyo, Japan; 3 Bayer Yakuhin Ltd., Osaka, Japan; 4 Bayer AG, Berlin, Germany; 5 Department of Ophthalmology, Kansai Medical University, Osaka, Japan

**Keywords:** Aflibercept, Exudative age-related macular degeneration, Ophthalmology, Treat-and-extend, Post-hoc

## Abstract

**Purpose:**

To explore the relationship between retinal fluid status and best-corrected visual acuity (BCVA) in patients treated with intravitreal aflibercept (IVT-AFL) treat-and-extend (T&E) in the ALTAIR study.

**Methods:**

Outcomes were investigated according to overall fluid status at week 16 (predefined) and the relationship between any fluid, intraretinal fluid (IRF), subretinal fluid (SRF), or pigment epithelial detachment with BCVA at baseline, and weeks 16, 52, and 96 (post-hoc). The analyses involved treatment-naïve patients (N = 246) with exudative age-related macular degeneration (AMD), aged ≥ 50 years with BCVA of 73–25 Early Treatment Diabetic Retinopathy Study letters, who participated in the ALTAIR study.

**Results:**

The mean (standard deviation) change in BCVA from baseline to week 52 was + 10.6 (10.9) and + 6.5 (16.0) letters in patients without and with fluid at week 16, respectively; and to week 96 was + 9.1 (14.3) and + 4.3 (16.1) letters in patients without and with fluid at week 16, respectively. The last injection interval was 16 weeks in 33.6% and 2.0% (week 52), and 62.9% and 17.6% (week 96) of patients without or with fluid at week 16, respectively. At baseline, 35.7% of patients had IRF and 85.2% of patients had SRF, which decreased to 11.8% (IRF) and 31.7% (SRF) of patients, 8.5% (IRF) and 18.7% (SRF), and 6.5% (IRF) and 20.7% (SRF) at weeks 16, 52, and 96, respectively. Presence of IRF at all timepoints was associated with poorer BCVA than if IRF was absent, while the presence of SRF was not associated with poorer BCVA compared with the absence of SRF.

**Conclusion:**

IVT-AFL T&E dosing was effective at clearing fluid regardless of fluid type in ~ 80% of patients with exudative AMD. Patients without fluid at week 16 had numerically better BCVA than those with fluid at week 16. Over 60% of patients without fluid at week 16 achieved the maximum treatment interval of 16 weeks by study end, compared with < 20% of patients with fluid at week 16. IRF (weeks 16, 52, 96), although evident in a small number of patients, was associated with poorer BCVA, whereas SRF was not.

**Trial registration:**

ClinicalTrials.gov Identifier: NCT02305238 
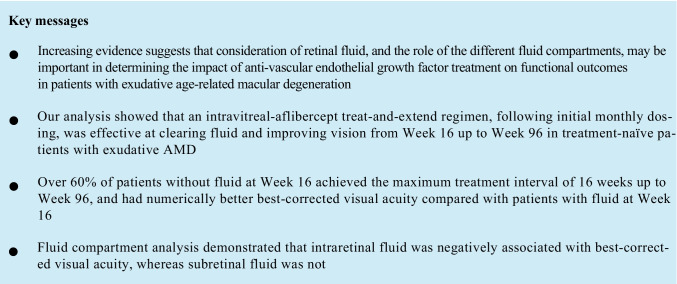

## Introduction

Exudative age-related macular degeneration (AMD) is characterized by abnormal growth of new blood vessels in the macula [1] and is the leading cause of AMD-related vision loss [2]. Clinical management of exudative AMD involves administering anti-vascular endothelial growth factor (VEGF) agents, such as aflibercept and ranibizumab [1], with the aim of improving functional and anatomic outcomes. However, anti-VEGF treatments are associated with clinic and patient-related burden, which can negatively impact long-term adherence and lead to increased healthcare costs [3].

Treat-and-extend (T&E) is a proactive, individualized dosing strategy whereby the treatment interval is gradually extended or shortened based on physician decision regarding the maintenance of functional and anatomic stability [4]. The flexibility offered with the T&E approach minimizes the risk of overtreatment and undertreatment, thus reducing treatment burden and the risk for disease recurrence, while optimizing functional and anatomic outcomes [3]. Results of the ALTAIR study demonstrated that, following initial monthly dosing, intravitreal aflibercept (IVT-AFL) administered in a T&E dosing regimen was effective in the first year of treatment and was continuously efficacious in the second year in patients with exudative AMD in Japan [5]. In the ALTAIR study, the criteria for injection interval shortening, maintenance, or extension were based on functional and anatomic outcomes (central retinal thickness [CRT], total fluid, intraretinal fluid [IRF], subretinal fluid [SRF], neovascularization, and hemorrhage) [5].

Increasing evidence suggests that consideration of fluid, and the role of the different compartments, may be important in determining the impact of anti-VEGF treatment on functional outcomes in patients with exudative AMD [6–8]. An analysis from the Comparison of AMD Treatments Trials (CATT), in which patients with exudative AMD were randomized to receive treatment with ranibizumab or bevacizumab on a monthly or as-needed schedule, reported that, at all timepoints, eyes with residual IRF (especially foveal IRF) had worse mean visual acuity (VA) than those without IRF, whereas eyes with SRF had better VA than those without SRF [6]. However, the relationship between retinal fluid status and functional outcomes is not well understood, and determining the impact of compartmental changes on functional outcomes following anti-VEGF treatment could help to optimize patient management. Given the lack of consensus regarding the impact of fluid status on functional outcomes, predefined and post-hoc analyses were conducted to explore baseline features that are potentially predictive of fluid status at week 16 and to investigate the relationship between overall retinal fluid status, fluid compartment type (IRF and SRF), and best-corrected visual acuity (BCVA) over 96 weeks in patients treated with IVT-AFL using an individualized T&E dosing regimen in the ALTAIR study.

## Methods

This article reports predefined and post-hoc exploratory analyses of data from ALTAIR, a 96-week, randomized, open-label, phase 4 study (ClinicalTrials.gov, NCT02305238) that was conducted to investigate the efficacy and safety of repeated doses of IVT-AFL with two different T&E approaches in patients with exudative AMD [5]. ALTAIR was conducted at 41 sites across Japan between December 2014 and November 2017, in accordance with the Declaration of Helsinki and the International Conference on Harmonization guidelines E6: Good Clinical Practice. The protocol was approved by the independent ethics committee or institutional review board at each study site. All patients provided written informed consent. As described in detail within the “Data analysis” section, the predefined analyses were conducted according to the presence or absence of fluid at week 16 and the presence or absence of fluid over time. The post-hoc analyses explored absolute BCVA by fluid status at each timepoint during the study.

### Study design

The methodology of the ALTAIR study has been published previously [5]. Patients received 2 mg IVT-AFL injections at baseline, week 4, week 8, and week 16. At week 16, patients were randomized 1:1 to T&E with the treatment interval adjusted in either 2-week (IVT-AFL-2 W) or 4-week (IVT-AFL-4 W) increments. Treatment intervals between IVT-AFL injections were extended, maintained, or shortened based on predefined functional and anatomic criteria; and the minimum and maximum treatment intervals were 8 and 16 weeks, respectively. The primary efficacy endpoint for the ALTAIR study was mean change in BCVA from baseline to week 52 [5]. Following the IVT-AFL injection at week 16, the timing of subsequent treatment visits was determined by the physician, based on predefined treatment criteria, at each visit. However, all patients were evaluated at weeks 52 and 96, regardless of treatment schedule.

The ALTAIR study involved treatment-naïve adults ≥ 50 years of age with exudative changes due to active subfoveal choroidal neovascularization lesions secondary to AMD, including juxtafoveal lesions that affected the fovea, as evidenced by fluorescein angiography in the study eye. Patients had a BCVA of 73–25 Early Treatment Diabetic Retinopathy Study (ETDRS) letters (approximately 20/40–20/320 Snellen equivalent) in the study eye.

### Study procedures

Data included in this analysis are based on optical coherence tomography (OCT) assessments generated during the ALTAIR study to guide treatment decisions. The OCT images were subsequently assessed by the investigator and the results recorded. The present analysis utilizes data based on the investigator assessments recorded during the ALTAIR study, and a separate analysis of OCT images was not conducted. Investigators were required to use spectral-domain OCT (SD-OCT; Supplementary Table 1) and the same machine was used throughout the study for follow-up of individual patients at each site.

### Data analysis

The aim of the analyses was to explore the relationship between any fluid (defined by presence of IRF and/or SRF; not including sub-retinal pigment epithelium fluid), as well as individual fluid compartments (IRF or SRF), and BCVA. Baseline demographics and disease characteristics according to fluid status at week 16 (with fluid/without fluid) were also explored.

These analyses comprise patients in the full analysis set (FAS; all randomized patients who received any study medication and had a baseline and ≥ 1 BCVA assessment after randomization [i.e., after week 16]); datasets for the IVT-AFL-2 W and IVT-AFL-4 W treatment arms were combined. The definitions of fluid used in the predefined analyses and the post-hoc analyses differed (as described in the “Predefined fluid analyses” and “Post-hoc analyses” sections). Statistical evaluation was performed using Statistical Analysis Software v9.4 (SAS Institute Inc., Cary, NC, USA).

#### Predefined fluid analyses

This analysis investigated overall fluid status on OCT assessment, defined as the presence or absence of any new or persistent fluid (remaining or increased fluid from the previous visit) at week 16. Treatment exposure, BCVA, and anatomic outcomes (mean change in BCVA and CRT) per week 16 fluid status were also explored, and 95% confidence intervals (CIs) were calculated. The proportion of patients with IRF and SRF was evaluated at each timepoint from baseline to week 96.

#### Post-hoc analyses

Baseline demographics and disease characteristics per fluid status (presence or absence of fluid) at week 16 were explored. A fluid compartment analysis was performed based on previously published methodology from CATT [6]. This analysis was designed to explore the relationship between any fluid (defined as IRF and/or SRF), IRF, SRF, or pigment epithelial detachment (PED) with BCVA at any timepoint (mandatory study visits occurred at baseline and weeks 16, 52, and 96). Fluid status, defined as the presence or absence of new or persistent fluid (IRF and/or SRF), was assessed using OCT (as described previously). If fluid was present, the location of the fluid relative to the foveal center was recorded as foveal (within 500 µm of the foveal center), or non-foveal (beyond 500 µm of the foveal center). Due to the exploratory nature of these analyses, descriptive statistical evaluation was conducted.

## Results

### Patients

All randomized patients in the ALTAIR study were included in these analyses (FAS, N = 246; IVT-AFL-2 W and IVT-AFL-4 W treatment arms combined). Information on baseline demographics and disease characteristics have previously been published [5]. Fluid status was unknown for 1 patient who had been randomized to the IVT-AFL-2 W group. In the predefined analysis, no fluid was observed on OCT conducted by the study investigators at week 16 in 58.1% (n = 148) of patients. In 41.5% (n = 102) of patients, the investigator noted presence of new or persistent fluid. Of patients without fluid at week 16, 85.3% and 84.6% had no fluid at weeks 52 and 96, respectively. Based on the predefined analysis, no fluid was recorded in 4.1% (n = 10/244) of patients at baseline and 58.1% (n = 143/246), 68.7% (n = 169/246), and 67.5% (n = 166/246) of patients at weeks 16, 52, and 96, respectively.

### Treatment exposure per week 16 fluid status

**Fig. 1:**
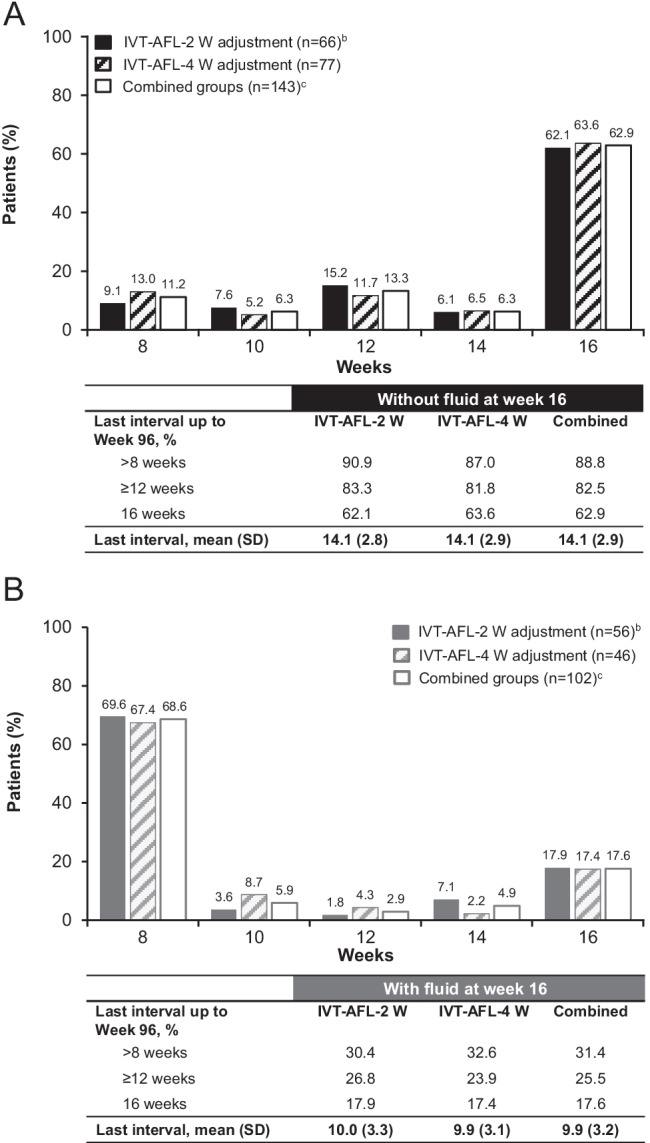
The proportion of patients with a last treatment interval up to week 96 of 8, 10, 12, 14, or 16 weeks and mean (SD) last treatment interval in **A** patients without fluid at week 16 and **B** patients with fluid at week 16 according to IVT-AFL adjustment regimen in the full analysis set (N = 246).^a,b,c^ The last treatment interval was calculated based on the last two doses received (overall, 227 patients completed week 52 of treatment and 212 patients completed week 96). ^a^Fluid status was assessed by the investigator based on the presence or absence of any new or persistent fluid (on OCT); ^b^Fluid status was unknown in the IVT-AFL-2 W adjustment group (n = 1); ^c^Two adjustment groups are combined (IVT-AFL-2 W and IVT-AFL-4 W).* IVT-AFL,* intravitreal aflibercept; *IVT-AFL-2 W,* IVT-AFL 2-week adjustment; *IVT-AFL-4 W,* IVT-AFL 4-week adjustment; *OCT,* optical coherence tomography; *SD,* standard deviation

Overall, from baseline to week 96, patients without and with fluid at week 16 received a mean (standard deviation [SD]) of 9.4 (1.7) and 11.8 (2.8) injections, respectively (Table 1). The mean (SD) last treatment interval at week 52 was 13.0 (2.9) and 8.9 (1.8) weeks, and at week 96 was 14.1 (2.9) and 9.9 (3.2) weeks in patients without and with fluid at week 16, respectively. The last injection interval before week 52 was ≥ 12 weeks in 67.1% (without fluid at week 16) and 16.7% (with fluid at week 16) of patients and before week 96 was ≥ 12 weeks in 82.5% (without fluid) and 25.5% (with fluid) of patients. The last injection interval before week 52 was 16 weeks in 33.6% (without fluid at week 16) and 2.0% (with fluid at week 16) of patients and before week 96 was 16 weeks in 62.9% (without fluid; Fig. 1A) and 17.6% (with fluid; Fig. 1B) of patients.

**Table 1:** Treatment exposure (number of IVT-AFL injections) per week 16 fluid status^a^

	Without fluid at week 16 (n = 143^b^)	With fluid at week 16 (n = 102^b^)
Baseline to week 96	9.4 (1.7)	11.8 (2.8)
Baseline to week 52	6.6 (0.8)	7.6 (1.0)
Week 52 to week 96^c^	3.0 (1.1)	4.7 (1.5)

Full analysis set (N = 246). All values are mean (SD)

^a^Fluid status was assessed by the investigator based on the presence of any new or persistent fluid (on OCT)

^b^Two adjustment groups are combined (IVT-AFL-2 W and IVT-AFL-4 W); fluid status was unknown in the IVT-AFL-2 W adjustment group (n = 1)

^c^Data are for weeks 52–96 in patients who were 52-week completers (without fluid [n = 135]; with fluid [n = 92])

*IVT-AFL*, intravitreal aflibercept; *IVT-AFL-2 W*, IVT-AFL 2-week adjustment; *IVT-AFL-4 W*, IVT-AFL 4-week adjustment; *OCT*, optical coherence tomography; *SD*, standard deviation

### BCVA and anatomic outcomes per week 16 fluid status

**Fig. 2:**
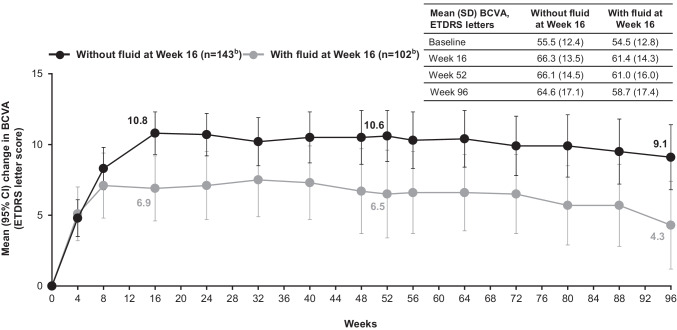
Mean (SD) absolute and mean (95% CI) change in BCVA (ETDRS letters) from baseline to week 96 in patients without and with fluid at week 16^a,b^ in the full analysis set (N = 246), with last observation carried forward. ^a^Fluid status was assessed by the investigator based on the presence or absence of any new or persistent fluid (on OCT); ^b^Two adjustment groups are combined (IVT-AFL-2 W and IVT-AFL-4 W); fluid status was unknown in the IVT-AFL-2 W group (n = 1). *BCVA,* best-corrected visual acuity; *CI,* confidence interval; *ETDRS,* Early Treatment Diabetic Retinopathy Study; *IVT-AFL,* intravitreal aflibercept; *IVT-AFL-2 W,* intravitreal aflibercept with 2-week adjustment; *IVT-AFL-4 W,* intravitreal aflibercept with 4-week adjustment; *OCT,* optical coherence tomography; *SD,* standard deviation

At baseline, mean (SD) BCVA was 55.5 (12.4) and 54.5 (12.8) letters for patients without and with fluid at week 16, respectively. For patients without fluid at week 16, the mean (SD) change in BCVA from baseline to week 52 was + 10.6 (10.9) letters, and to week 96 was + 9.1 (14.3) letters. For patients with fluid at week 16, the mean change in BCVA was + 6.5 (16.0) letters and + 4.3 (16.1) letters from baseline to weeks 52 and 96, respectively (Fig. 2).

**Fig. 3:**
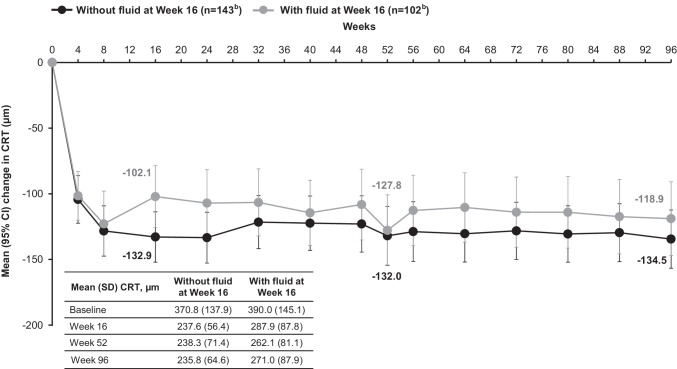
Mean (SD) absolute and mean (95% CI) change in CRT (μm) from baseline to week 96 in patients without and with fluid at week 16^a,b^ in the full analysis set (N = 246), with last observation carried forward. ^a^Fluid status was assessed by the investigator based on the presence or absence of any new or persistent fluid (on OCT); ^b^Two adjustment groups are combined (IVT-AFL-2 W and IVT-AFL-4 W); fluid status was unknown in the IVT-AFL-2 W group (n = 1). *CI,* confidence interval; *CRT,* central retinal thickness; *IVT-AFL,* intravitreal aflibercept; *IVT-AFL-2 W,* intravitreal aflibercept with 2-week adjustment; *IVT-AFL-4 W,* intravitreal aflibercept with 4-week adjustment; *OCT,* optical coherence tomography; *SD,* standard deviation

At baseline, mean (SD) CRT was 370.8 (137.9) µm and 390.0 (145.1) µm for patients without and with fluid at week 16, respectively. For patients without fluid at week 16, the mean (SD) change in CRT from baseline to week 52 was − 132.0 (135.3), and to week 96 was − 134.5 (133.8) µm. For patients with fluid at week 16, the mean change in CRT was − 127.8 (138.9) and − 118.9 (144.4) µm from baseline to weeks 52 and 96, respectively (Fig. 3).

### Baseline features per week 16 fluid status: a post-hoc analysis

Post-hoc exploration of baseline features suggested that patients with a relatively thick CRT, a high PED height, who did not have polypoidal choroidal vasculopathy (PCV), or who did not have a subretinal hemorrhage were more likely to have retinal fluid at week 16 (Table 2). Mean (SD) baseline CRT was 370.8 (137.9) µm (patients without fluid at week 16) and 390.0 (145.1) µm (patients with fluid at week 16). At baseline, PED was observed in 55.9% of patients without fluid at week 16 (mean PED height: 271.1 µm), compared with 78.4% of patients with fluid at week 16 (mean PED height: 341.4 µm). At baseline, a total of 41.3% of patients had PCV among those without fluid at week 16, compared with 29.4% of those with fluid at week 16.

**Table 2:** Baseline features per week 16 fluid status^a^

	Without fluid at week 16 (n = 143^b^)	With fluid at week 16 (n = 102^b^)
Age, years	74.3 (8.2)	73.6 (7.9)
Male, n (%)	102 (71.3)	75 (73.5)
BCVA, ETDRS letters	55.5 (12.4)	54.5 (12.8)
CRT, µm	370.8 (137.9)	390.0 (145.1)
Type of AMD,^c^ n (%)		
Typical AMD	80 (55.9)	70 (68.6)
PCV	59 (41.3)^d^	30 (29.4)^d^
RAP	9 (6.3)^d^	4 (3.9)^d^
Type of CNV lesions on FA,^e^ n (%)		
Classic	51 (35.7)	26 (25.5)
Classic and occult	20 (14.0)	11 (10.8)
Occult	70 (49.0)	63 (61.8)
No CNV	1 (0.7)	0 (0.0)
IRF, n (%)	54 (37.8)^f^	33 (32.4)
SRF, n (%)	116 (81.1)^g^	91 (89.2)
PED, n (%)	80 (55.9)^g^	80 (78.4)
Mean height of PED, µm	271.1 (179.2)	341.4 (266.8)
Mean width of PED, µm	2293.1 (1231.5)	2370.1 (1553.9)
Subretinal hemorrhage, n (%)	72 (50.3)^d^	33 (32.4)^g^
Intraretinal hemorrhage, n (%)	39 (27.3)^d^	19 (18.6)^g^

Full analysis set (N = 246). All values are mean (SD) unless otherwise stated

^a^Fluid status was assessed by the investigator based on the presence of any new or persistent fluid (on OCT)

^b^Two adjustment groups are combined (IVT-AFL-2 W and IVT-AFL-4 W); fluid status was unknown in the IVT-AFL-2 W group (n = 1)

^c^Some patients were diagnosed as 1 or 2 subtypes

^d^Missing (n = 1)

^e^Unknown for patients without fluid (n = 1) and with fluid (n = 2)

^f^Missing (n = 2), unknown (n = 3)

^g^Missing (n = 2)

*AMD*, age-related macular degeneration; *BCVA*, best-corrected visual acuity; *CNV*, choroidal neovascularization; *CRT*, central retinal thickness; *ETDRS*, Early Treatment Diabetic Retinopathy Study; *FA*, fluorescein angiography; *IRF*, intraretinal fluid; *PCV*, polypoidal choroidal vasculopathy; *PED*, pigment epithelial detachment; *RAP*, retinal angiomatous proliferation; *SRF*, subretinal fluid

### Fluid compartment analysis

**Fig. 4:**
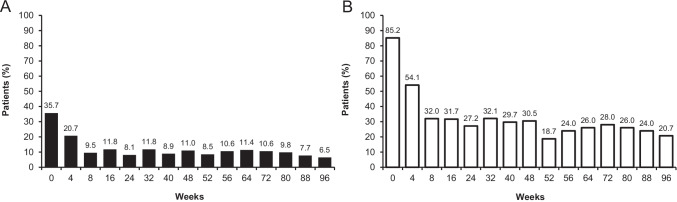
The proportion of patients with **A** IRF or **B** SRF at each timepoint from baseline to week 96^a,b^ in the full analysis set (N = 246), with last observation carried forward. ^a^Fluid status was assessed by the investigator based on the presence or absence of any new or persistent fluid (on OCT); ^b^Two adjustment groups are combined (IVT-AFL-2 W and IVT-AFL-4 W); IRF and SRF fluid status was unknown in n = 2 at baseline and in n = 5 at week 8. *IRF,* intraretinal fluid; *IVT-AFL,* intravitreal aflibercept; *IVT-AFL-2 W,* intravitreal aflibercept with 2-week adjustment; *IVT-AFL-4 W,* intravitreal aflibercept with 4-week adjustment; *OCT,* optical coherence tomography; *SRF,* subretinal fluid

In the predefined fluid compartment analysis, over 70% of patients had no new or persistent IRF or SRF at any measured timepoint after week 8 (Fig. 4). At baseline, 35.7% and 85.2% of patients had IRF or SRF, which had reduced by week 16 to 11.8% and 31.7% of patients for the respective fluid compartment.

### BCVA according to fluid compartment: a post-hoc analysis

**Fig. 5:**
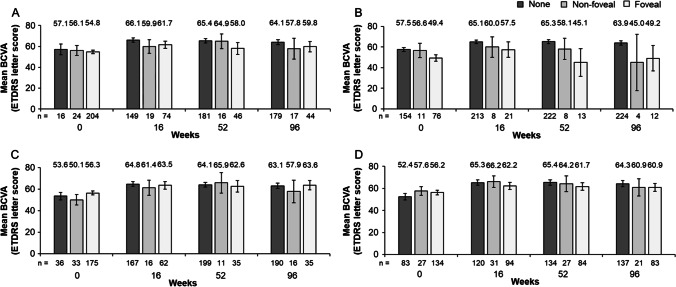
Mean absolute BCVA (ETDRS letters) and 95% confidence intervals at mandatory study visits at weeks 0, 16, 52, and 96 in **A** patients without or with any foveal or non-foveal fluid,^a^ **B** patients without or with any foveal or non-foveal IRF, **C** patients without or with any foveal or non-foveal SRF, and **D** patients without or with any foveal or non-foveal PED. Full analysis set (N = 246).^b,c^ The number underneath each bar represents the number of patients in each group at each study visit. ^a^Includes IRF and SRF only; ^b^Missing for each fluid compartment at baseline (n = 2); ^c^Unknown for IRF at baseline (n = 3). *BCVA,* best-corrected visual acuity; *ETDRS,* Early Treatment Diabetic Retinopathy Study; *IRF,* intraretinal fluid; *PED,* pigment epithelial detachment; *SRF,* subretinal fluid

In this post-hoc analysis, mean BCVA (ETDRS letters) was 57.1 at baseline (n = 16), 66.1 at week 16 (n = 149), 65.4 at week 52 (n = 181), and 64.1 at week 96 (n = 179) for patients without any fluid (Fig. 5A).^1^ The presence of foveal IRF at all timepoints was associated with lower BCVA (ETDRS letters) than if IRF was absent (foveal IRF vs no IRF: 49.4 vs 57.5 at baseline; 57.5 vs 65.1 at week 16; 45.1 vs 65.3 at week 52; and 49.2 vs 63.9 letters at week 96). A similar trend was observed with non-foveal IRF compared with no IRF at baseline, week 16, and week 52 (Fig. 5B). Conversely, the presence of foveal SRF was not associated with poorer BCVA (ETDRS letters) compared with the absence of SRF (Fig. 5C). A similar trend was observed with non-foveal SRF compared with no SRF at all timepoints (Fig. 5C). Any adverse effects of the presence of foveal PED on BCVA (Fig. 5D) appeared to be relatively small (< 5 letters).

SRF was not associated with lower BCVA values, unless IRF was also present (defined as the presence or absence of IRF at week 52; data not shown). Overall, 33 patients had new and/or persistent SRF both at weeks 16 and 52. Of these, 30 patients did not have coexisting IRF at week 52, and, in this group, the mean BCVA was maintained from week 16 (65.5 letters [SD; range: 13.4; 19.0–86.0]) to week 96 (64.1 letters [13.9; 19.0–83.0]). Of the 33 patients with new and/or persistent SRF both at weeks 16 and 52, 3 patients had coexisting IRF at week 52, and, in this group, the mean BCVA decreased from week 16 (63.7 letters [4.0; 60.0–68.0]) to week 96 (51.3 letters [1.5; 50.0–53.0]).

## Discussion

In this predefined fluid analysis of data from the ALTAIR study, IVT-AFL T&E dosing was effective at clearing fluid and improving vision by week 16 in treatment-naïve patients with exudative AMD. The mean BCVA was similar between groups at baseline, and the mean change to week 52 was + 10.6 letters and + 6.5 letters in patients without and with fluid at week 16, respectively, with outcomes maintained to week 96. The mean change in CRT from baseline to week 96 followed a similar pattern; however, at baseline, there was a slight difference between groups (370.8 µm and 390.0 µm in patients without and with fluid at week 16, respectively). Regarding the last treatment interval up to week 96, over 60% of patients without fluid at week 16 achieved the maximum treatment extension interval of 16 weeks, compared with 17.6% of patients with fluid at week 16. From baseline to week 96, patients without and with fluid at week 16 received a mean of 9.4 and 11.8 injections of IVT-AFL, respectively. The post-hoc evaluation of baseline features suggested that patients with relatively thinner CRT and a lower PED height at baseline were more likely to have no fluid at week 16. A higher proportion of patients without fluid at week 16 had PCV and subretinal hemorrhage at baseline than those with fluid at week 16. Previous studies involving patients with PCV have reported comparable visual outcomes to those with typical AMD following treatment with IVT-AFL fixed or T&E dosing [9, 10]. This will be investigated further in future analyses.

Overall, the fluid compartment analysis showed that IVT-AFL T&E dosing was effective at clearing fluid in ~ 80% of patients with exudative AMD over the 96-week treatment period and led to improved BCVA, regardless of the fluid type. Over 70% of patients had no new or persistent IRF or SRF at any measured timepoint after week 8, following the three initial doses of IVT-AFL. The role of the different fluid compartments in determining the impact of anti-VEGF treatment on functional outcomes in patients with exudative AMD is a growing area of interest [6–8]. In the CATT analysis, eyes with residual IRF (especially foveal IRF) had worse VA than those without IRF, whereas eyes with SRF had better VA than those without SRF [6]; findings were replicated at 2 and 5 years [7, 8]. In the 5-year CATT analysis, foveal IRF was independently associated with worse VA over the course of the study, and patients with foveal SRF had better VA at 5 years than those without SRF [8]. Findings from the present CATT-like post-hoc analysis add to the evidence, demonstrating that new or persistent foveal IRF, although evident in a small number of patients, was associated with lower BCVA. The reasons for the specific potential adverse impact of IRF on BCVA are not well understood, and suggestions include that intraretinal cystoid fluid may be a sign of a more aggressive lesion type as well as a sign of late presentation in chronic occult choroidal neovascularization (CNV) [11]. IRF accumulates predominantly in the Henle fiber layer and the inner nuclear layer of the retina [12]. IRF likely results from damage to the outer blood–retinal barrier and to external limiting membrane integrity, thus allowing subretinal exudation to enter the outer retinal layers, from photoreceptors through to the Henle fiber layer. Subsequent loss of photoreceptors could lead to decreased vision.

In our analysis, conducted in patients treated with IVT-AFL, new or persistent SRF (foveal or non-foveal) was not associated with lower BCVA, consistent with previous reports [6–8]. However, the effect of persistent SRF on VA is unclear. In our further analysis, of the 33 patients with new and/or persistent SRF both at weeks 16 and 52, 3 patients had coexisting IRF at week 52, and, in this group, the mean BCVA decreased from 63.7 letters (week 16) to 51.3 letters (week 96); however, because of the small number of patients, it is difficult to draw conclusions. The effects of coexisting IRF and SRF on BCVA remain unclear; therefore, further investigation is warranted.

The present analysis explored the relationship between fluid status at week 16 after initial dosing and BCVA following treatment with IVT-AFL in a T&E regimen over 2 years, suggesting that absence of fluid at week 16 was predictive of good outcomes. In the ALTAIR study [5], patients received three initial monthly doses of IVT-AFL (weeks 0, 4, 8), and because week 16 was the last visit before randomization, it was the optimal timepoint to evaluate retinal fluid status following similar initial dosing in all patients. Onward from week 16 was considered to be the beginning of the maintenance phase of treatment with IVT-AFL. The positive impact of early reductions in retinal fluid with IVT-AFL treatment has been reported previously with a monthly dosing regimen in the VIEW trials. VA improvements from baseline in eyes with early persistent fluid were observed by week 16 and were maintained to week 52 with monthly injections of IVT-AFL. The pattern of functional outcomes was similar regardless of fluid type (IRF or SRF) [13]. Approximately 60% of patients had their fluid resolved by week 16. While 40% of patients still had persistent fluid at this timepoint, > 30% of these patients had been able to extend treatment intervals beyond 8 weeks. Further investigation is needed to understand the clinical relevance of persistent fluid in the respective compartments, and a differentiated approach to adapt treatment intervals should be formulated with the goal of maintaining improvements in visual function.

The main strength of this study was that the data were obtained from a large randomized trial with a strict protocol, offering the opportunity to explore the relationship between fluid status and BCVA/anatomic outcomes in patients with exudative AMD. Furthermore, to our knowledge, this was the first CATT-like fluid analysis in patients treated with IVT-AFL. We should acknowledge that the CATT analysis identified various other factors (e.g., presence of subretinal hyper-reflective material, thinner retina, and greater CNV lesion area [8]) as being independently associated with BCVA loss. It was not possible to investigate these in our own analysis; however, the relationship between fluid and BCVA is valid despite the potential contributions of other drivers.

A limitation of these analyses was the differing definitions of fluid between the predefined analysis, which was based on the presence or absence of new or persistent fluid, and the post-hoc analysis, which was based on the presence or absence of any fluid (specifically IRF and/or SRF). Furthermore, the original study was not designed to determine the relationships between compartment fluid status and impact of disease outcomes after IVT-AFL treatment, and as the specific volume of fluid was not evaluated, quantitative fluid analysis could not be conducted. Limitations in relation to SD-OCT techniques and SD-OCT scan analysis for the determination of fluid status must also be acknowledged. Although there was no reading center involved in the ALTAIR study, SD-OCT machine and image settings for OCT were the same throughout the study at each site. The SD-OCT images were not read by the same observer nor were they checked by another individual, but were guided by a specific OCT manual.

In conclusion, this analysis of data from the ALTAIR study demonstrated that IVT-AFL T&E dosing was effective at clearing fluid in over 70% of treatment-naïve patients with exudative AMD and improving vision. Findings from the predefined analysis demonstrated that patients without fluid had better BCVA than those with fluid at week 16. In patients with exudative AMD, baseline features such as CRT and PED height may play a role in determining the initial response of retinal fluid to treatment with IVT-AFL; further research is required to investigate predictive factors for response. The post-hoc analysis showed that IRF, although evident in a small number of patients, was associated with lower BCVA, whereas foveal SRF was not. Further quantitative analyses are required to confirm these findings.

## Data Availability

Availability of the data underlying this publication will be determined according to Bayer’s commitment to the EFPIA/PhRMA, “Principles for responsible clinical trial data sharing.” This pertains to scope, timepoint, and process of data access. As such, Bayer commits to sharing, upon request from qualified scientific and medical researchers, patient-level clinical trial data, study-level clinical trial data, and protocols from clinical trials in patients for medicines and indications approved in the USA and European Union (EU) as necessary for conducting legitimate research. This applies to data on new medicines and indications that have been approved by the EU and US regulatory agencies on or after January 1, 2014. Interested researchers can use www.clinicalstudydatarequest.com to request access to anonymized patient-level data and supporting documents from clinical studies to conduct further research that can help advance medical science or improve patient care. Information on the Bayer criteria for listing studies and other relevant information is provided in the study sponsors section of the portal. Data access will be granted to anonymized patient-level data, protocols, and clinical study reports after approval by an independent scientific review panel. Bayer is not involved in the decisions made by the independent review panel. Bayer will take all necessary measures to ensure that patient privacy is safeguarded.

## Footnotes

1 Fluid refers to IRF and SRF compartments.
