# Correction to: Relationship between retinal fluid and visual acuity in patients with exudative age-related macular degeneration treated with intravitreal aflibercept using a treat-and-extend regimen: subgroup and post-hoc analyses from the ALTAIR study

**DOI:** 10.1007/s00417-022-05621-w

**Published:** 2022-04-02

**Authors:** Masahito Ohji, Annabelle A. Okada, Koji Sasaki, SungChul Charles Moon, Tobias Machewitz, Kanji Takahashi, on behalf of the ALTAIR Investigators

**Affiliations:** 1 Department of Ophthalmology, Shiga University of Medical Science, Shiga, Japan; 2 Department of Ophthalmology, Kyorin University School of Medicine, Tokyo, Japan; 3 Bayer Yakuhin Ltd., Osaka, Japan; 4 Bayer AG, Berlin, Germany; 5 Department of Ophthalmology, Kansai Medical University, Osaka, Japan

**Correction to: Graefe’s Archive for Clinical and Experimental Ophthalmology (2021) 259:3637–3647**

**https://doi.org/10.1007/s00417-021-05293-y**

The original version of this article unfortunately contained errors. The corrections are given in the following list:

1. In Fig. 5A, the patient group with “Any fluid” at the non-foveal location incorrectly also included a few patients whose fluid presence is unknown. The corresponding non-foveal data have been re-evaluated and Fig. 5A amended. Specifically, data for the number of patients, mean BCVA, and 95% confidence intervals have been updated for the bars representing non-foveal fluid at weeks 16, 52, and 96. The correct data are now presented in Fig. 5A.
2. In Fig. 5, it was also not specified that the footnotes “Missing for each fluid compartment (*n* = 2)” and “Unknown for IRF (*n* = 3)” were at baseline (not at other timepoints). The Fig. 5 footnote has now been corrected to clarify that these cases were at baseline.

**Fig. 1:**
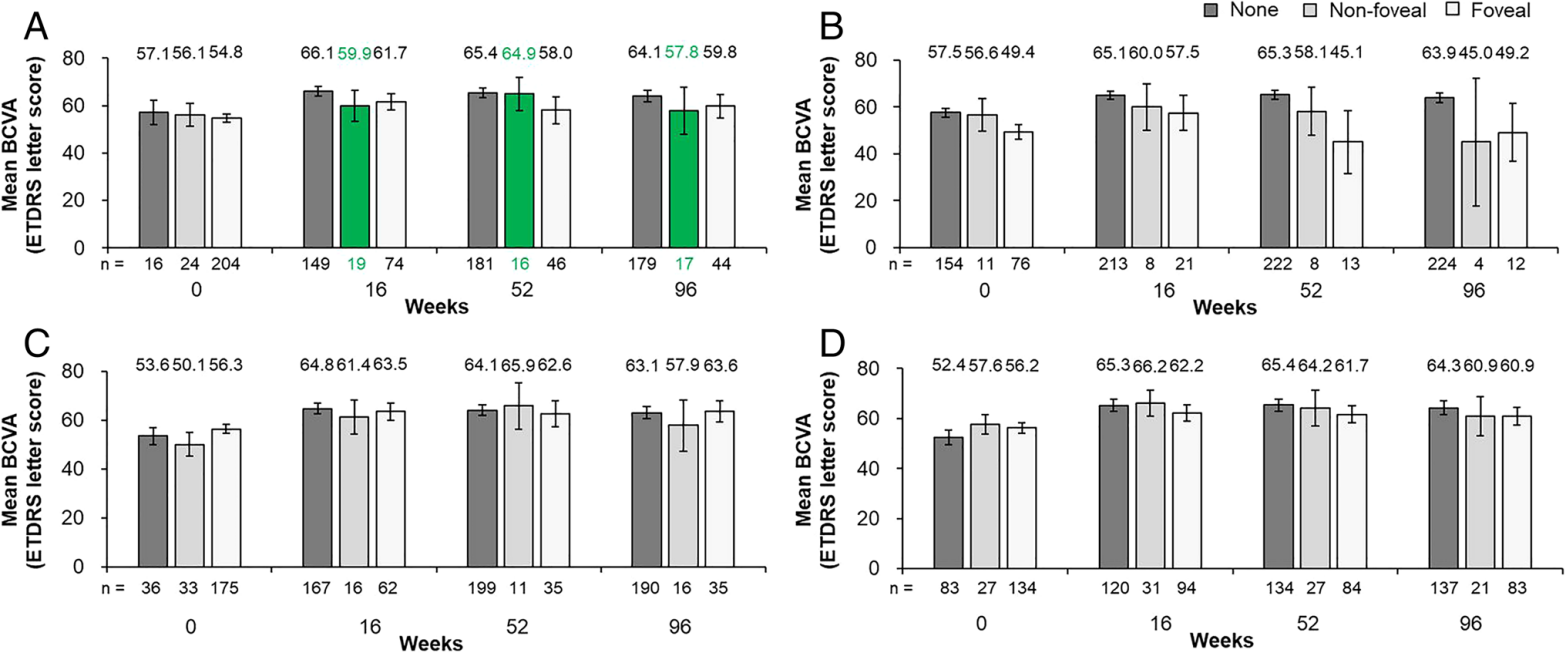
Mean absolute BCVA (ETDRS letters) and 95% confidence intervals at mandatory study visits at weeks 0, 16, 52, and 96 in **A** patients without or with any foveal or non-foveal fluid,^a^ **B** patients without or with any foveal or non-foveal IRF, **C** patients without or with any foveal or non-foveal SRF, and **D** patients without or with any foveal or non-foveal PED. Full analysis set (*N* = 246).^b,c^ The number underneath each bar represents the number of patients in each group at each study visit. ^a^Includes IRF and SRF only; ^b^Missing for each fluid compartment  (*n* = 2); ^c^Unknown for IRF  (*n* = 3). *BCVA*, best-corrected visual acuity; *ETDRS*, Early Treatment Diabetic Retinopathy Study; *IRF*, intraretinal fluid; *PED*, pigment epithelial detachment; *SRF*, subretinal fluid

The correct figure and legend are shown below. Revisions are shown in green for ease of reference.

This is being corrected in this publication.

